# Hemoglobin Analysis in the First Year of Life

**DOI:** 10.4084/MJHID.2016.012

**Published:** 2016-02-12

**Authors:** Peerapon Wong, Jiranun Weerakul, Suchila Sritippayawan

**Affiliations:** 1 Thalassemia Research Unit, Faculty of Medicine, Naresuan University, Phitsanulok, Thailand; 2 Department of Pediatrics, Faculty of Medicine, Naresuan University, Phitsanulok, Thailand; 3 Department of Obstetrics and Gynecology, Faculty of Medicine, Naresuan University, Phitsanulok, Thailand

## Abstract

**Background and Objectives:**

In newborns and infants during their first year of life, there is a dynamic change in the fraction of hemoglobin (Hb). To apply Hb analysis as a phenotypic diagnosis of thalassemia in newborns and infants, we need normal values of each Hb fraction for reference.

**Methods:**

Seventeen cord bloods from normal deliveries were collected for analysis. One hundred and thirty-seven infants from the pediatric outpatient clinic were recruited and were categorized by their ages into a series of short periods (month±2 weeks). Both alpha and beta thalassemia carriers detected were excluded. Samples with an Hb level less than 10.0 g/dL were also excluded. The proportion of Hb A (α_2_β_2_), A_2_ (α_2_δ_2_), and F (α_2_γ_2_) was obtained from high-performance liquid chromatography and analyzed according to its categorized periods.

**Results:**

There were 90 (58.4%) specimens left for evaluation. The percentage of Hb A, A_2_, and F gradually changed with increasing age. The percentage of Hb A was 21.14±7.04% (mean±SD) in cord blood and increased substantially to 83.38±1.31% at the sixth month. The level was sustained thereafter. The incremental pattern of Hb A_2_ was similar to Hb A. The value was 0.32±0.19% at the beginning and reached a plateau with 2.78±0.25% at the sixth month. The percentage of Hb F started at 78.39±7.59% in cord blood and decreased rapidly in the first 6 months.

**Conclusions:**

The data possibly can be applied as quick guidance for interpretation of Hb analysis in newborns and infants during their first year of life.

## Introduction

Thalassemia is the most common genetic disorder in Southeast Asia. Screening and diagnosis for at-risk couples and performing prenatal diagnosis (PND) for affected fetuses are the practical strategies to cope with this problem. In mass screening and diagnosis in adults, hemoglobin (Hb) analysis plays a major role. By using the proportion and relative quantity of the normal Hb fraction, namely Hb A (α_2_β_2_), A_2_ (α_2_δ_2_), and F (α_2_γ_2_), the thalassemia phenotype can be identified leading to a further specific DNA method for final genotypic diagnosis. Besides the thalassemia prevention and control program in adults, in some special situations, parents may seek investigation for their newborn baby regarding thalassemia. Whether for finding diagnosis of anemia etiology or for reassurance of their thalassemia status after prior PND, Hb analysis is also usually undertaken. In newborns and infants during their first year of life, there is a dynamic change in the fraction of Hb, making their Hb analyzes totally different from the results for adults. Steady increment of beta (β) globin chain synthesis is inverse to the reduction of gamma (γ) globin chain production. As a result, the quantity of Hb A constantly climbs while Hb F keeps falling. In adults, the normal proportion of Hb A_2_ is 2.5–3.5%.^1^ When this value is exceeded (> 3.5%),^1^,^2^ the diagnosis of beta-thalassemia carrier can then be established. However, since a small quantity of the delta (δ) globin chain gradually increases in newborns, the proportion of Hb A_2_ is constantly lower than for adults, making the diagnosis of beta-thalassemia carrier impossible when using the Hb cutoff value for adults. Therefore, when applying the Hb analysis as a screening tool to narrow down further the genotypic diagnosis in newborns and infants during their first year of life, we need normal values of the relative quantity of the Hb fraction at this age. Available information regarding newborn Hb has some limitations in clinical practice. One report provides only the proportion of Hb F by alkaline denaturation technique and has been classified only in some specific points of time.^3^ Another one provides the proportion of Hb A_2_ by capillary isoelectric focusing technique in only 3 age groups: 5 months or younger, 6 months to 1 year, and 1 year or older.^4^ However, in real life situations, the newborns may come in any specific point of time, and all fractions of Hb together are needed to help in interpreting their thalassemia phenotypes. In addition, these Hb analyzes should also be performed with a current method being used worldwide. In conclusion, our study’s objective was to provide indicative values of Hb A, A_2_, and F by using high-performance liquid chromatography (HPLC) and categorize the results by a series of time periods during the newborns and infants’ development.

## Materials and Methods

A prospective study was conducted between July 2010 and April 2011. One hundred and thirty-seven blood specimens from infants during their first year of life from the pediatric outpatient clinic and 17 cord bloods from normal deliveries were consecutively collected at Naresuan University Hospital, Phitsanulok, Thailand. Each infant attended the clinic for any diseases which needed their blood checked. Blood specimens were collected for the study in parallel with their needed investigation. One hundred and thirty-seven infants consecutively recruited were categorized by age into a series of short periods. These were the first, the second, the third, the fourth, the fifth, the sixth, the eighth, the tenth, and the twelfth month±2 weeks. The proportion of Hb A, A_2_, and F was obtained from HPLC (VARIANT ), using the β-thalassemia Short Program (Bio-Rad Laboratories, California, USA), in each sample and analyzed according to its categorized periods into mean±SD. Each Hb value was directly calculated from the machine without modification. The polymerase chain reaction (PCR) to detect the alpha thalassemia-1 (Southeast Asian and Thai deletions) and alpha thalassemia-2 (3.7 kb and 4.2 kb deletions) genotype was also performed in every case. Together with alpha thalassemia PCR, detection of 6 common beta-thalassemia mutations (codon 41/42 (-TTCT), codon 17 (A-T), IVS-I nt1 (G-T), IVS-I nt5 (G-C), codon 71/72 (+A), and codon 26 (G-A) or Hb E) was conducted using the multiplex amplification refractory mutation system (ARMS).^5^,^6^ Both alpha and beta thalassemia carriers detected were excluded from the study. Complete blood count was performed in all specimens. Samples with an Hb level less than 10 g/dL were also excluded. Relative quantities of the Hb fraction from the excluded thalassemia carriers were used to compare with the indicative values created. The study was approved by the institutional ethics committee (5302040017). Written informed consent was obtained from all parents before entering the study.

## Results

**Figure 1:**
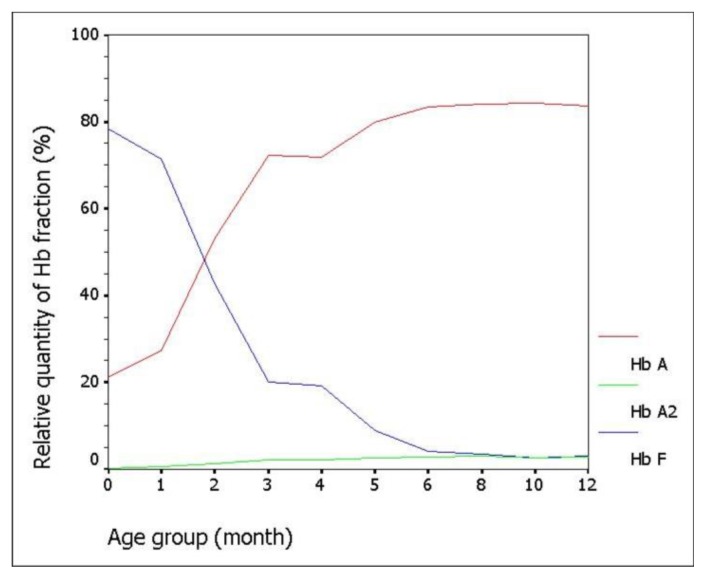
The dynamic change of the proportion of hemoglobin A, A_2_, and F of the newborns and infants within the first year of life

Among 154 samples, after exclusion of thalassemia carriers, there were 100 (64.9%) specimens left: 10 cord bloods and 90 samples from infants. Of these infant samples, there were 10 with Hb levels less than 10.0 g/dL and had to be further excluded. Gestational age for the 10 cord bloods ranged from 37.3 weeks to 40.1 weeks (mean 38.7±0.8 weeks). Among the 80 evaluable infants attending the pediatric outpatient clinic, there were 66 (82.5%) with infections and 14 (17.5%) with non-infectious diseases. The infectious diseases comprised 25 gastroenteritis, 19 lower respiratory tract infections, 14 upper respiratory tract infections, and 8 other infections. The non-infectious illnesses were 4 conjugated hyperbilirubinemias, 3 unconjugated hyperbilirubinemias, 3 patent ductus arteriosus, 2 hydrocephalus, and 2 other causes. The proportion of Hb A, A_2_, and F analyzed according to the categorized periods is shown in Table 1 and Figure 1. The sum of other non-specific minor peaks (P_1_, P_2_, and P_3_) is also provided (Table 1).

For the 54 thalassemia carriers excluded, there were 17 alpha thalassemia-2 heterozygotes, 13 Hb E heterozygotes, 10 alpha thalassemia-1 heterozygotes, 7 Hb E and alpha thalassemia-2 double heterozygotes, 2 Hb E and alpha thalassemia-1 double heterozygotes, 1 beta thalassemia and alpha thalassemia-1 double heterozygote, 1 beta thalassemia compound heterozygote, 2 alpha thalassemia-2 homozygotes and 1 Hb E homozygote. When we compared the Hb analyzes of these thalassemia carriers with our indicative values according to their age groups, some guidance for the phenotypic interpretation could be seen (Table 2). Hb A_2_ and Hb E are generally eluted from HPLC with the same retention time but in different amounts. All 23 samples with Hb E related genotype had a high proportion of Hb A_2_/Hb E, out of range (> 2 SD) from each categorized period. Lower Hb F and A_2_ values were observed in the majority of alpha thalassemia-1 heterozygote. However, these values were still in our indicative ranges. No phenotypic differences could be detected in newborns with alpha thalassemia-2 heterozygote. One beta thalassemia and alpha thalassemia-1 double heterozygote had high Hb A_2_ value out of our indicative range without beta thalassemia mutation initially identified, using our multiplex ARMS. However, with further DNA investigation, codon 27/28 (+C) was eventually detected. One beta thalassemia compound heterozygote had high Hb F level without Hb A. Also, beta thalassemia mutations [codon 17 (AT) and codon 26 (G-T)] were finally identified using DNA sequencing method.

## Discussion

Because of its well-accepted high accuracy and our center’s experience, HPLC was the method selected in this study for determining the Hb value. The percentage of Hb A, A_2_, and F gradually changed with increasing age according to normal development. Hb A and A_2_ kept rising with age, inverse to the proportion of Hb F. The percentage of Hb A was 21.14±7.04% (mean±SD) in cord blood and increased substantially to 83.38±1.31% at the sixth month. The level was sustained thereafter. The incremental pattern of Hb A_2_ was similar to Hb A. The value was 0.32±0.19% at the beginning and reached a plateau with 2.78±0.25% at the sixth month. The percentage of Hb F started at 78.39±7.59% in cord blood and decreased rapidly in the first 6 months. The lowest value was around 3% at the tenth and the twelfth month. In the first 6 months of life after birth, there is a more dynamic change in the fraction of Hb compared with the period after 6 months, reflected by high SD values in each categorized period. Age difference in weeks or even in days can affect the more relative quantity of each Hb fraction in this earlier period. Compared with available information provided in the literature,^3^,^4^ there are some differences of values in the details, which may be due to the different techniques performed and different periods categorized. Otherwise, there could be regional and ethnic variations in the percentage of Hb. Compared with previously published values since the mid-1980s using the alkaline denaturation technique (Table 3),^3^ the percentage of Hb F after 8 months of age seems higher in our study which may be due to the accuracy of HPLC used.^7^ However, the major trend of Hb F reduction from previous data looks the same as ours. Compared with the other report in which the value of Hb A_2_ of the age group from 6 months to 1 year was 2.2±0.9%,^4^ the percentage of Hb A_2_ in every categorized period after 6 months of age seems higher in our study with less variation (SD value) (Table 4). This difference may be due to the different technique performed.^8^,^9^ The value of Hb A_2_ of the age group less than 5 months from the same report was 1.2±1.5% which cannot be compared with our data which had more periods categorized.

As we compared the relative quantities of the Hb fraction from thalassemia carriers with our indicative Hb values, there were some differences usable for phenotypic interpretation, as in adults. All samples with the Hb E related genotype could be obviously identified from each categorized period with high Hb E level. As in adults, no apparent differences could be detected in newborns with alpha thalassemia-1 or alpha thalassemia-2 heterozygote, despite slightly lower Hb F and A_2_ values observed in alpha thalassemia-1 heterozygote. In beta thalassemia heterozygote, higher Hb A_2_ level could be detected as in adults. However, we still did not have enough data to see the true differences. The clinical application of our indicative Hb values could be more obvious when used to compare samples with thalassemia disease. There would be no Hb A detected in the beta thalassemia homozygote, compound heterozygote or Hb E/beta thalassemia compound heterozygote, as indicated in one of our thalassemia samples.

For the limitation of the study, some possible confounders which can affect the proportion of each Hb cannot totally be excluded. The six beta thalassemia mutations tested can cover 91.8%, but not all of the beta globin mutations in the North of Thailand.^6^ In addition, DNA analysis to detect alpha-Hb Constant Spring was not performed. The study did not rule out iron deficiency which can theoretically reduce alpha globin chain synthesis and affect the quantity of the Hb fraction, especially Hb A_2_.^10^,^11^ However, more recent studies found this effect as minimal, and it might be negligible.^12^–^14^ Infection and underlying disease of the infants recruited can also in part suppress the level, but not the proportion, of their Hb depending on the severity of their illnesses.^15^ This limitation is directly due to ethical issues. It seems inappropriate to collect blood specimens from healthy infants for research without needed investigation or any benefit. Therefore, we chose to recruit infants as healthy as possible from the outpatient clinic. Nevertheless, to make sure there was no anemic condition to affect the level of each Hb fraction, we also excluded samples with Hb levels less than 10.0 g/dL, which is the lower limit (-2 SD) accepted for normal Hb in the infants.^16^

The results of Hb A, A_2_, and F of newborns, and infants within the first year of life, in our study, are more practical in details for specific age, separated by month, of an infant compared with the available information. The thalassemia genotype of samples recruited, which was the most important confounding factor, was substantially excluded. However, among the limitations of the study are the relatively small number of infants and the diversity of illnesses from the outpatient samples recruited. With these limitations, the observed Hb percentages can only be considered as indicative and not as reference values. Nevertheless, the data possibly can be applied as quick guidance for interpretation of Hb analysis in patients during their first year of life.

## Figures and Tables

**Table 1:** Hemoglobin (Hb) level and proportion of Hb A, A_2_, F, and other non-specific minor peaks (P_1_, P_2_, and P_3_) of the newborns and infants within the first year of life

Categorized period	N	Hb (g/dL)	Hb A (%)	Hb A_2_ (%)	Hb F (%)	Minor peaks (%)
At birth/cord blood	10	15.25±2.56	21.14±7.04	0.32±0.19	78.39±7.59	0.67±0.92
**1**^st^ month±**2** week	10	12.64±1.75	27.20±14.20	0.55±0.68	71.49±16.03	1.28±1.17
**2**^nd^ month±**2** week	8	11.03±0.64	53.04±13.93	1.40±0.54	42.79±15.36	2.78±1.22
**3**^rd^ month±**2** week	10	10.84±0.76	72.23±8.11	2.14±0.47	20.04±11.32	5.59±3.03
**4**^th^ month±**2** week	7	11.27±0.63	71.73±9.49	2.09±0.57	19.19±11.74	7.00±3.34
**5**^th^ month±**2** week	11	11.65±1.02	79.91±6.52	2.65±0.37	9.01±7.54	8.43±1.26
**6**^th^ month±**2** week	8	11.28±0.68	83.38±1.31	2.78±0.25	4.08±1.62	9.78±0.97
**8**^th^ month±**2** week	6	11.23±0.38	84.13±1.72	2.98±0.17	3.47±1.92	9.42±0.84
**10**^th^ month±**2** week	9	12.14±1.03	84.38±1.46	2.67±0.21	2.53±1.58	10.42±1.32
**12**^th^ month±**2** week	11	11.58±0.89	83.67±1.76	2.78±0.20	3.16±1.86	10.38±1.44

Values shown as mean±SD. Abbreviations: Hb, hemoglobin

**Table 2:** Proportion of hemoglobin A, A_2,_ and F of the newborns and infants within the first year of life comparing between normal and thalassemia carrier.

Categorized period	Genotype	N	Hb A (%)	Hb A_2_/E (%)	Hb F (%)
At birth/cord blood	β^A^/β^A^, αα/αα	10	21.14±7.04	0.32±0.19	78.39±7.59
	β^E^/β^A^, αα/αα	2	5.80±1.27	3.40±1.56	90.0±1.98
	β^A^/β^A^, _ _^SEA^/αα	2	24.90±5.37	0.15±0.21	74.30±6.36
	β^A^/β^A^, _^3.7^α/αα	2	22.30±11.74	0.55±0.21	76.25±11.10
	β^A^/β^A^, _^3.7^α/_^3.7^α	1	28.50	0.40	69.70
1^st^ month±2 week	β^A^/β^A^, αα/αα	10	27.20±14.20	0.55±0.68	71.49±16.03
	β^E^/β^A^, αα/αα	2	17.50±9.76	7.25±4.17	75.20±14.57
	β^A^/β^A^, _ _^SEA^/αα	1	47.60	1.00	50.00
	β^A^/β^A^, _^3.7^α/αα	3	24.73±11.88	0.37±0.40	73.57±12.56
	β^E^/β^A^, _ _^SEA^/αα	1	26.70	6.30	62.10
	β^E^/β^A^, _^3.7^α/αα	2	25.70±1.98	6.70±4.24	65.80±2.40
2^nd^ month±2 week	β^A^/β^A^, αα/αα	8	53.04±13.93	1.40±0.54	42.79±15.36
	β^E^/β^A^, αα/αα	2	24.45±19.59	10.40±7.35	63.75±27.65
	β^A^/β^A^, _^3.7^α/αα	1	43.10	1.20	53.10
3^rd^ month±2 week	β^A^/β^A^, αα/αα	10	72.23±8.11	2.14±0.47	20.04±11.32
	β^A^/β^A^, _^3.7^α/αα	1	76.20	2.30	14.80
	β^E^/β^A^, _^3.7^α/αα	1	44.10	19.10	34.90
4^th^ month±2 week	β^A^/β^A^, αα/αα	7	71.73±9.49	2.09±0.57	19.19±11.74
	β^E^/β^A^, αα/αα	1	54.50	25.30	12.30
	β^E^/β^A^, _^3.7^α/αα	1	60.10	22.10	9.10
5^th^ month±2 week	β^A^/β^A^, αα/αα	11	79.91±6.52	2.65±0.37	9.01±7.54
	β^E^/β^A^, αα/αα	1	57.00	21.80	5.50
	β^A^/β^A^, _ _^SEA^/αα	2	83.25±1.20	2.55±0.07	4.35±0.64
6^th^ month±2 week	β^A^/β^A^, αα/αα	8	83.38±1.31	2.78±0.25	4.08±1.62
	β^E^/β^A^, αα/αα	1	59.80	23.90	4.50
	β^A^/β^A^, _ _^SEA^/αα	2	78.90±3.25	2.20±0.00	4.95±3.18
	β^A^/β^A^, _^3.7^α/αα	4	82.43±1.69	2.63±0.17	4.85±2.51
	β^E^/β^A^, _ _^SEA^/αα	1	54.30	6.30	9.30
	β^E^/β^A^, _^4.2^α/αα	1	61.10	17.30	6.00
8^th^ month±2 week	β^A^/β^A^, αα/αα	6	84.13±1.72	2.98±0.17	3.47±1.92
	β^E^/β^A^, αα/αα	1	56.80	22.30	17.20
	β^A^/β^A^, _ _^SEA^/αα	2	81.80±3.39	2.45±0.35	6.10±4.80
	β^A^/β^A^, _^3.7^α/αα	3	81.83±1.97	2.67±0.15	4.87±2.10
	β^E^/β^A^, _^3.7^α/αα	1	60.90	24.10	7.20
	β^0^/β^A^, _ _^SEA^/αα	1	65.10	4.70	5.50
	β^0^/β^0^, αα/αα	1	0.70	3.00	99.70
10^th^ month±2 week	β^A^/β^A^, αα/αα	9	84.38±1.46	2.67±0.21	2.53±1.58
	β^A^/β^A^, _ _^SEA^/αα	1	83.00	2.60	1.90
	β^A^/β^A^, _^3.7^α/αα	1	85.50	2.70	1.20
	β^A^/β^A^, _^3.7^α/_^3.7^α	1	81.0	2.80	6.10
12^th^ month±2 week	β^A^/β^A^, αα/αα	11	83.67±1.76	2.78±0.20	3.16±1.86
	β^E^/β^A^, αα/αα	3	66.03±8.58	20.07±6.59	3.10±1.49
	β^A^/β^A^, _^3.7^α/αα	2	84.25±1.06	2.80±0.14	1.75±0.07
	β^E^/β^A^, _^3.7^α/αα	1	64.00	25.00	2.40
	β^E^/β^E^, αα/αα	1	8.60	75.90	11.10

Values shown as mean±SD. Abbreviations: Hb, hemoglobin; β^A^/β^A^, αα/αα, normal genotype; β^E^/β^A^, αα/αα, Hb E heterozygote; β^A^/β^A^, _ _^SEA^/αα, alpha thalassemia-1 (Southeast Asian deletion) heterozygote; β^A^/β^A^, _^3.7^α/αα, alpha thalassemia-2 (3.7 kb deletion) heterozygote; β^E^/β^A^, _ _^SEA^/αα, Hb E and alpha thalassemia-1 double heterozygote; β^E^/β^A^, _^3.7^α/αα, Hb E and alpha thalassemia-2 (3.7 kb deletion) double heterozygote; β^E^/β^A^, _^4.2^α/αα, Hb E and alpha thalassemia-2 (4.2 kb deletion) double heterozygote; β^0^/β^A^, _ _^SEA^/αα, beta thalassemia and alpha thalassemia-1 double heterozygote; β^0^/β^0^, αα/αα, beta thalassemia homozygote or compound heterozygote; β^A^/β^A^, _^3.7^α/_^3.7^α, alpha thalassemia-2 (3.7 kb deletion) homozygote; β^E^/β^E^, αα/αα, Hb E homozygote

**Table 3:** Proportion of hemoglobin (Hb) F of the newborns and infants within the first year of life comparing between our study and a previously published study^3^

Categorized period from our study	Hb F (%)	Categorized period from previous study^3^	Hb F (%)*
At birth/cord blood	78.39±7.59	1 day	77.0±7.3
		5 days	76.8±5.8
1^st^ month±2 week	71.49±16.03	3 weeks	70.0±7.3
2^nd^ month±2 week	42.79±15.36	6–9 weeks	52.9±11.0
3^rd^ month±2 week	20.04±11.32	3–4 months	23.2±16.0
4^th^ month±2 week	19.19±11.74		
5^th^ month±2 week	9.01±7.54		
6^th^ month±2 week	4.08±1.62	6 months	4.7±2.2
8^th^ month±2 week	3.47±1.92	8–11 months	1.6±1.0
10^th^ month±2 week	2.53±1.58		
12^th^ month±2 week	3.16±1.86		

Values shown as mean±SD.

* Percentage of Hb F was measured by alkaline denaturation technique.

Abbreviations: Hb, hemoglobin

**Table 4:** Proportion of hemoglobin (Hb) A_2_ of the newborns and infants within the first year of life comparing between our study and a previously published study^4^

Categorized period from our study	Hb A_2_ (%)	Categorized period from previous study^4^	Hb A_2_ (%)*
At birth/cord blood	0.32±0.19	5 months or younger	1.2±1.5
1^st^ month±2 week	0.55±0.68		
2^nd^ month±2 week	1.40±0.54		
3^rd^ month±2 week	2.14±0.47		
4^th^ month±2 week	2.09±0.57		
5^th^ month±2 week	2.65±0.37		
6^th^ month±2 week	2.78±0.25	6 months to 1 year	2.2±0.9
8^th^ month±2 week	2.98±0.17		
10^th^ month±2 week	2.67±0.21		
12^th^ month±2 week	2.78±0.20		

Values shown as mean±SD.

* Percentage of Hb A_2_ was measured by capillary isoelectric focusing technique.

Abbreviations: Hb, hemoglobin
